# Serum CA 15-3 assay in the diagnosis and follow-up of breast cancer.

**DOI:** 10.1038/bjc.1988.196

**Published:** 1988-08

**Authors:** O. P. Kallioniemi, H. Oksa, R. K. Aaran, T. Hietanen, M. Lehtinen, T. Koivula

**Affiliations:** Department of Clinical Chemistry, Tampere University Central Hospital, Finland.

## Abstract

Serum CA 15-3 values were determined in 177 patients with primary breast cancer and in 41 with non-malignant breast disease. Increased preoperative serum CA 15-3 values (greater than 38 U ml-1) were observed in 7%, 17%, 64% and 67% of patients with stage I, II, III and IV disease, respectively and in none of the patients with benign breast disease. Patients with elevated serum CA 15-3 values had poor 3-year cumulative survival (27%). In the postoperative follow-up 9% of patients with no clinical evidence of disease, 33% with a single metastasis and 67% with two or more metastases had elevated values. Increasing or decreasing serum CA 15-3 values correlated with the clinical outcome in 26 out of 27 cases (96%), whereas serum values remaining in the reference range had no predictive value. At the time of recurrence elevated serum CA 15-3 values were also observed in patients with normal preoperative values. Increased serum CA 15-3 values preceded the clinical detection of tumour recurrence by up to 13 months. In conclusion, serum CA 15-3 levels had prognostic value in breast cancer, reflected the extent of clinically detectable disease and the presence of occult metastatic disease. Further research is warranted on the benefits of CA 15-3 assays in relation to adjuvant chemotherapy as well as the earlier detection and treatment of metastatic disease.


*Br. J. Cancer* (1988), **58**, 213–215 © The Macmillan Press Ltd., 1988

### Serum CA 15-3 assay in the diagnosis and follow-up of breast cancer

O-P. Kallioniemi[1], H. Oksa[2], R-K. Aaran[3], T. Hietanen[2], M. Lehtinen[3] & T. Koivula[1]

[1]*Department of Clinical Chemistry, Tampere University Central Hospital, SF-33520 Tampere; and Departments of* [2]*Clinical and* [3]*Biomedical Sciences, University of Tampere, Tampere, Finland.*

**Summary** Serum CA 15-3 values were determined in 177 patients with primary breast cancer and in 41 with non-malignant breast disease. Increased preoperative serum CA 15-3 values ($>38\,U\,ml^{-1}$) were observed in 7%, 17%, 64% and 67% of patients with stage I, II, III and IV disease, respectively and in none of the patients with benign breast disease. Patients with elevated serum CA 15-3 values had poor 3-year cumulative survival (27%). In the postoperative follow-up 9% of patients with no clinical evidence of disease, 33% with a single metastasis and 67% with two or more metastases had elevated values. Increasing or decreasing serum CA 15-3 values correlated with the clinical outcome in 26 out of 27 cases (96%), whereas serum values remaining in the reference range had no predictive value. At the time of recurrence elevated serum CA 15-3 values were also observed in patients with normal preoperative values. Increased serum CA 15-3 values preceeded the clinical detection of tumour recurrence by up to 13 months. In conclusion, serum CA 15-3 levels had prognostic value in breast cancer, reflected the extent of clinically detectable disease and the presence of occult metastatic disease. Further research is warranted on the benefits of CA 15-3 assays in relation to adjuvant chemotherapy as well as the earlier detection and treatment of metastatic disease.

The value of clinical laboratory tests in the follow-up of breast cancer patients has been questioned due to their insensitivity and nonspecificity (Ormiston *et al.*, 1985; Tomin & Donegan, 1987). CEA has been the only tumour marker suitable in some cases for the monitoring of treatment in advanced breast cancer (Tormey & Waalkes, 1978; Mughal *et al.*, 1983). More sensitive and specific serum markers are required in the detection of tumour recurrence and in the monitoring of treatment response.

A radioimmunometric assay based on two different monoclonal antibodies (115 D8 and DF3) has been introduced for a breast cancer-associated antigen CA 15-3 (Kufe *et al.*, 1984; Hilkens *et al.*, 1984). Preliminary clinical evidence indicates that in the follow-up of breast cancer CA 15-3 is more sensitive than CEA (Hayes *et al.*, 1986; Würz *et al.*, 1986; Pons-Anicet *et al.*, 1987). In the present study we evaluated the sensitivity and specificity of CA 15-3 and the use of this assay in the follow-up of breast cancer patients.

### Materials and methods

Serum samples were collected from 177 patients with primary breast cancer and from 58 age-adjusted patients with nonmalignant breast disease. All patients were operated in the Tampere University Central Hospital in 1981–1985. Both preoperative and postoperative (1–3 year follow-up) serum samples were obtained from 68 patients. The samples were stored at −70°C for 1–6 years before analysis. The patients were staged according to the TNM classification. In the postoperative period judgement of the presence or absence of tumour was based on clinical examination, chest and bone radiographs and in a few cases isotope scans.

Serum CA 15-3 levels were determined using an immunoradiometric assay kit (ELSA-CA 15-3, International CIS, Cedex, France). The reference range for serum CA 15-3 level was determined on the basis of values obtained from patients with non-malignant breast diseases (Mean + 3 s.d.).

### Results

The upper reference value for normal CA 15-3 value was set at $38\,U\,ml^{-1}$. In the preoperative period, markedly elevated serum CA 15-3 values were observed mainly in patients with locally advanced (stage III) and metastatic (stage IV) breast cancer (Figure 1). None of the patients with benign breast tumours or breast infections had elevated serum values. Short-term follow-up (median 1.9 years) of the patients indicated 27% cumulative 3-year survival for cases with elevated preoperative serum CA 15-3 levels as compared to 84% for patients with normal serum levels ($P<0.001$, Mantel–Cox test). Since this analysis was based on small numbers of patients with elevated values and short follow-up time it was not possible to determine whether CA 15-3 values had prognostic value independent of the TNM staging.

In serum samples obtained 1–5 years after operation CA 15-3 again showed a good correlation with clinical disease status (Figure 2). About two-thirds of the patients with several metastases had elevated serum CA 15-3 values. One-third of the patients with a single (in most cases locoregional) metastasis and only 9% of patients with no clinical evidence of disease had increased serum values. One patient had markedly elevated serum CA 15-3 value without any clinical evidence of disease (Figure 2). At the time of blood sampling this patient apparently had subclinical disease because 13 months later an intra-abdominal breast cancer metastasis was diagnosed (Figure 3).

Follow-up of the patients disclosed that serum values remaining in the reference range did not give reliable infor-

**Figure 1** Preoperative serum CA 15-3 values in patients with breast cancer and other non-malignant breast diseases. The percentage of patients having increased serum values is shown.

Correspondence: O-P. Kallioniemi.
Received 30 November 1987; and in revised form, 19 April 1988.

**Figure 2** Serum CA 15-3 values 1—5 years after operation according to clinical disease status. The percentage of patients with elevated serum values is shown.

**Figure 4** Relation of serum CA 15-3 levels with disease course in a patient with primary breast cancer. Serum CA 15-3 levels raised concomitantly with the detection of multiple bone, liver and lung metastasis (Dg), which rapidly lead to the death (D) of the patient.

**Table I** Relation of disease course to changes in serum CA 15-3 levels. If only those patients who at some stage of the disease had elevated serum CA 15-3 levels were included in the table (figures in parentheses), changes in serum CA 15-3 levels paralleled clinical disease course in 26/27 cases (96%)

| | *Serum CA 15-3 values* | | |
|---|---|---|---|
| | *Remain in the reference range* | *Increase* | *Decrease* |
| No evidence of cancer | 19 (0) | – | – |
| Progression of cancer | 21 (1) | 19 (19) | – |
| Regression of cancer | – | – | 7 (7) |

**Figure 3** Relation of serum CA 15-3 levels with disease course in a patient operated for primary breast cancer. Elevated serum CA 15-3 values preceeded the clinical diagnosis (Dg) of intra-abdominal carcinomatosis by 13 months. Following combination chemotherapy and antiestrogen treatment (Th) complete clinical response was obtained with concomitant decrease of serum CA 15-3 levels to the reference range.

mation on changes of tumour mass of the patients. However, if only those patients with increasing or decreasing serum levels were considered, changes in serum CA 15-3 concentration paralleled the clinical course of the disease in 26 out of 27 (96%) cases (Table I) during a 1–3 year follow-up period (Figures 3 and 4). At the time of recurrence elevated serum CA 15-3 values were also observed in patients with normal preoperative values.

### Discussion

In patients with various breast diseases CA 15-3 values higher than 38 U ml$^{-1}$ appeared to be highly specific for cancer. Previous investigators have reported elevated values ranging from 5% (Würz *et al.*, 1986) to 22% (Hayes *et al.*, 1986) in benign breast diseases, using 30 U ml$^{-1}$ as the cut-off level. Hayes *et al.* (1986) have also detected elevated values in 1/16 (6%) of lactating women and in 20–80% of malignancies other than breast cancer. In our study the higher cut-off value was based on the analysis of samples from patients with benign breast diseases and enabled us to achieve higher specificity without notable decrease in sensitivity.

Both the present and previous investigations (Würz *et al.*, 1986; Pons-Anicet *et al.*, 1987) indicate that increased pre-operative values of CA 15-3 are observed mainly in patients with advanced breast cancer and not in the more common early-stage tumours. Therefore, the sensitivity of the assay does not allow use of CA 15-3 as a sole diagnostic or prognostic test for breast cancer. The present preliminary results indicated that elevated preoperative values may have prognostic significance. Whether serum CA 15-3 values add independent information to the prognostic assessment of breast cancer or merely reflect tumour burden similar to TNM-staging remains to be determined. It would be advantageous to incorporate measurements of serum CA 15-3 levels in adjuvant therapy trials. In this regard the critical issue is, whether serum CA 15-3 values are raised in micro-metastatic disease, or only in advanced macrometastatic disease, where adjuvant therapy is no more indicated. On the basis of the clinicopathological correlations of CA 15-3, the latter possibility appears more likely, although only a follow-up study of stage I–II breast cancer patients with elevated preoperative serum CA 15-3 levels will give data in this respect.

At the time of metastasis elevated serum CA 15-3 values were also observed in patients who had normal preoperative levels. In fact, about two-thirds of patients with more than one metastasis had elevated values. It thus seems that in the postoperative monitoring of disease recurrence all patients and not only those with elevated preoperative values may benefit from this assay. However, both the present and previous (Hayes *et al.*, 1986; Würz *et al.*, 1986; Pons-Anicet *et al.*, 1987) results suggest that serum CA 15-3 assay is not very sensitive in detecting a single locoregional metastasis.

In the follow-up of the patients increasing and decreasing serum CA 15-3 values have more clinical significance than values remaining in the reference range. It should also be noted that patients may have progressing disease in spite of persistently low serum CA 15-3 levels. In the present study we demonstrated that serum CA 15-3 values may be elevated up to 13 months prior to the clinical diagnosis of residive. Apparently disease-free patients with elevated serum CA 15-3 values may therefore have occult metastasis. Due to the slow disease progression in breast cancer, with metastases occurring up to 15–20 years (Sutherland & Mather, 1986; Harris & Hellman, 1986) after initial treatment, the chances of detecting such subclinical metastases are considerable. However, at the time being there is no evidence indicating that the earlier detection of metastatic disease by serial tumour marker assays is beneficial in terms of gained survival time. Further research on subclinical metastatic breast disease and CA 15-3 levels is warranted before elevated serum levels could justify immediate therapeutic intervention.

This study was supported by grants from the Finnish Cancer Society and the Kaija Ahonen Fund of the Pirkanmaa Cultural Foundation.

### References

HARRIS, J.R. & HELLMAN, S. (1986). Observations on survival curve analysis with particular reference to breast cancer treatment. *Cancer*, **57**, 925.

HAYES, D.F., ZURAWSKI, V.R. & KUFE, D.W. (1986). Comparison of circulating CA 15-3 and carcinoembryonic antigen levels in patients with breast cancer. *J. Clin. Oncol.*, **4**, 1542.

HILKENS, J., HILGERS, J., BUIJS, F. & 4 others (1984). Monoclonal antibodies against human milk fat globule membranes useful in carcinoma research. In *Protides of Biological Fluids*, Peeters, H. (ed) **Vol. 31**, p. 1013. Pergamon Press: New York.

KUFE, D., INGHIRAMI, G., ABE, M., HAYES, D., JUSTIWHEELER, H. & SCHOLM, J. (1984). Differential reactivity of a monoclonal antibody (DF3) with human malignant versus benign breast tumors. *Hybridoma*, **3**, 223.

MUGHAL, A.W., HORTOBAGYI, G.N., FRITSCHE, H.A., BUZDAR, A.V., YAP, H.Y. & BLUMENSCHEIN, G.R. (1983). Serial plasma carcinoembryonic antigen measurements during treatment of metastatic breast cancer. *J. Amer. Med. Assoc.*, **249**, 1881.

ORMISTON, M.C., TIMONEY, A.G., QURESHI, A.R. (1985). Is follow-up of patients after surgery for breast cancer worthwhile? *J. Royal Soc. Med.*, **78**, 920.

PONS-ANICET, D.M.F., KREBS, B.P. & NAMER, M. (1987). Value of CA 15-3 in the follow-up of breast cancer patients. *Br. J. Cancer*, **55**, 567.

SUTHERLAND, C.M. & MATHER, F.J. (1986). Long-term survival and prognostic factors in breast cancer patients with localized (no skin, muscle or chest wall attachment) disease with and without positive lymph nodes. *Cancer*, **57**, 622.

TOMIN, R. & DONEGAN, W. (1987). Screening for recurrent breast cancer – Its effectiveness and prognostic value. *J. Clin. Oncol.*, **5**, 62.

TORMEY, D.C. & WAALKES, T.P. (1978). Clinical correlation between CEA and breast cancer. *Cancer*, **42**, 1507.

WÜRZ, H. & CROMBACH, G. (1986). Clinical evaluation of tumor associated antigen CA 15-3 in breast cancer. *XIV Ann. Mtg. Int. Soc. Oncodevelop. Biol. & Med.*, Helsinki 1986 (*Abstract No. 83*).


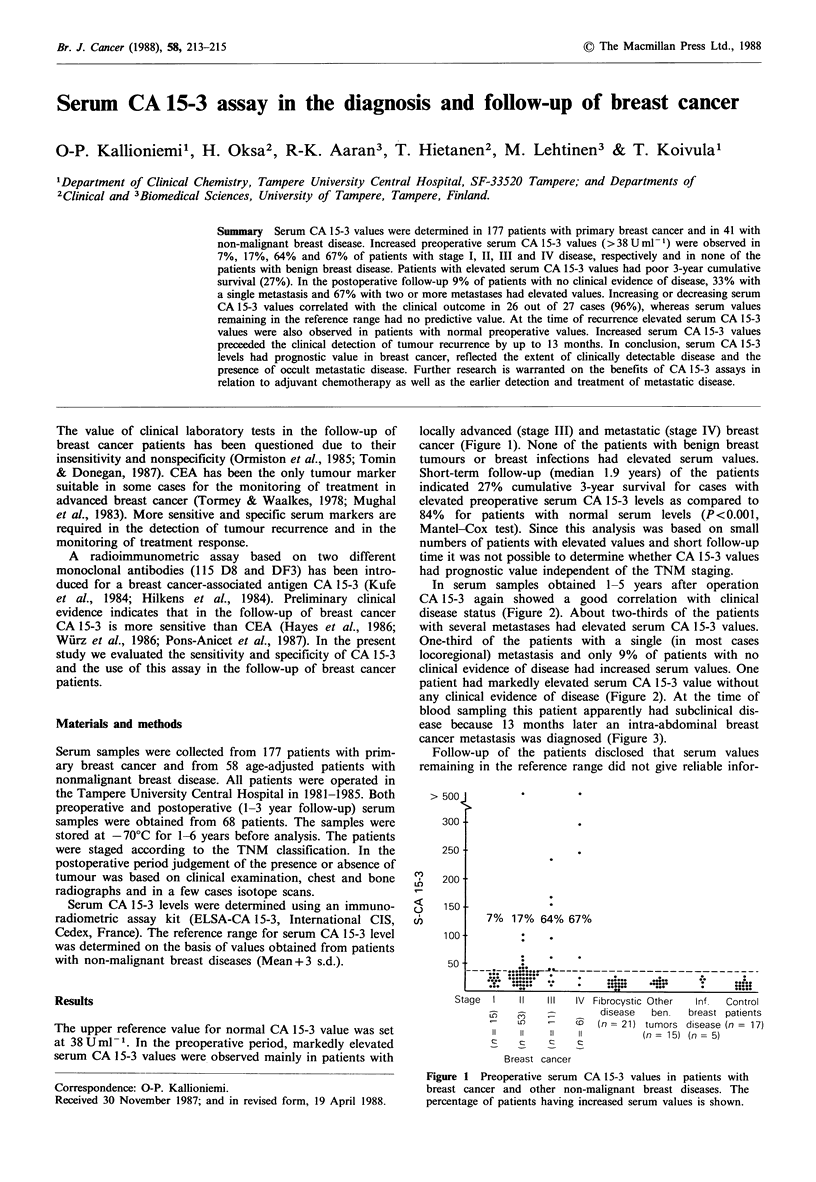

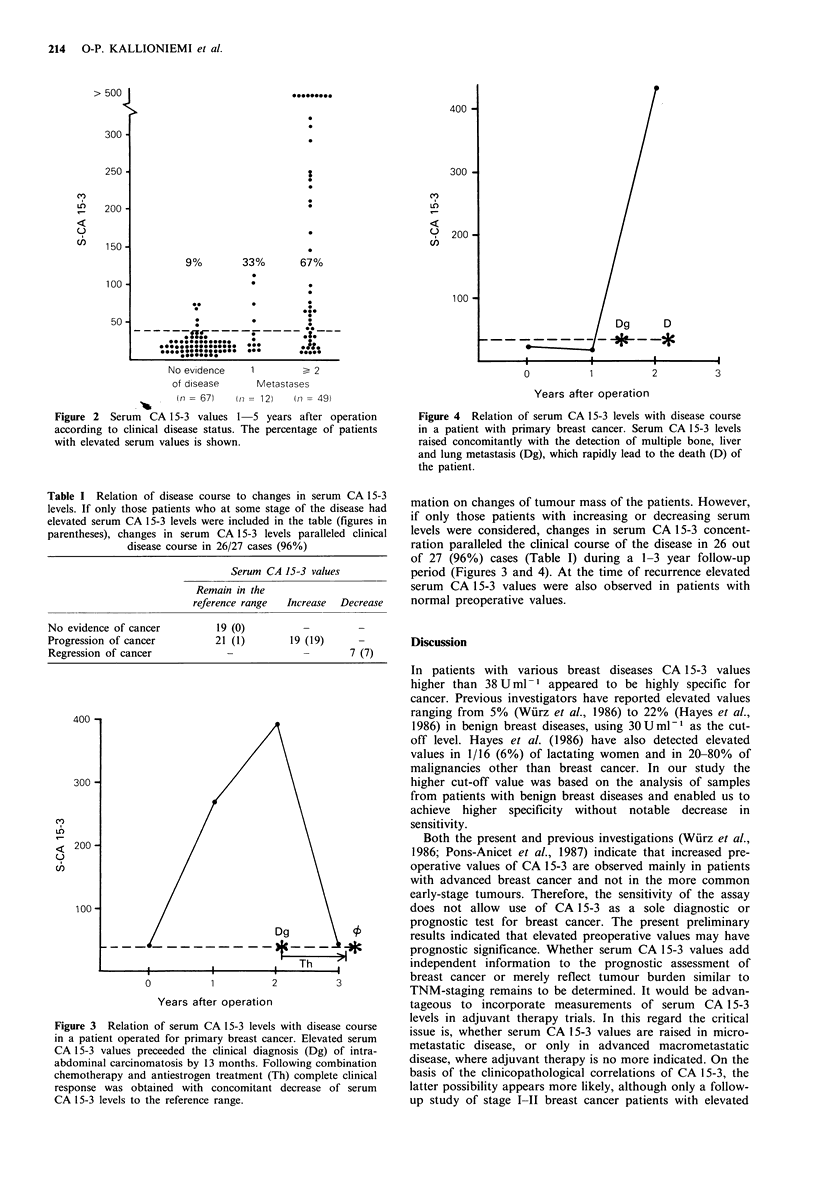

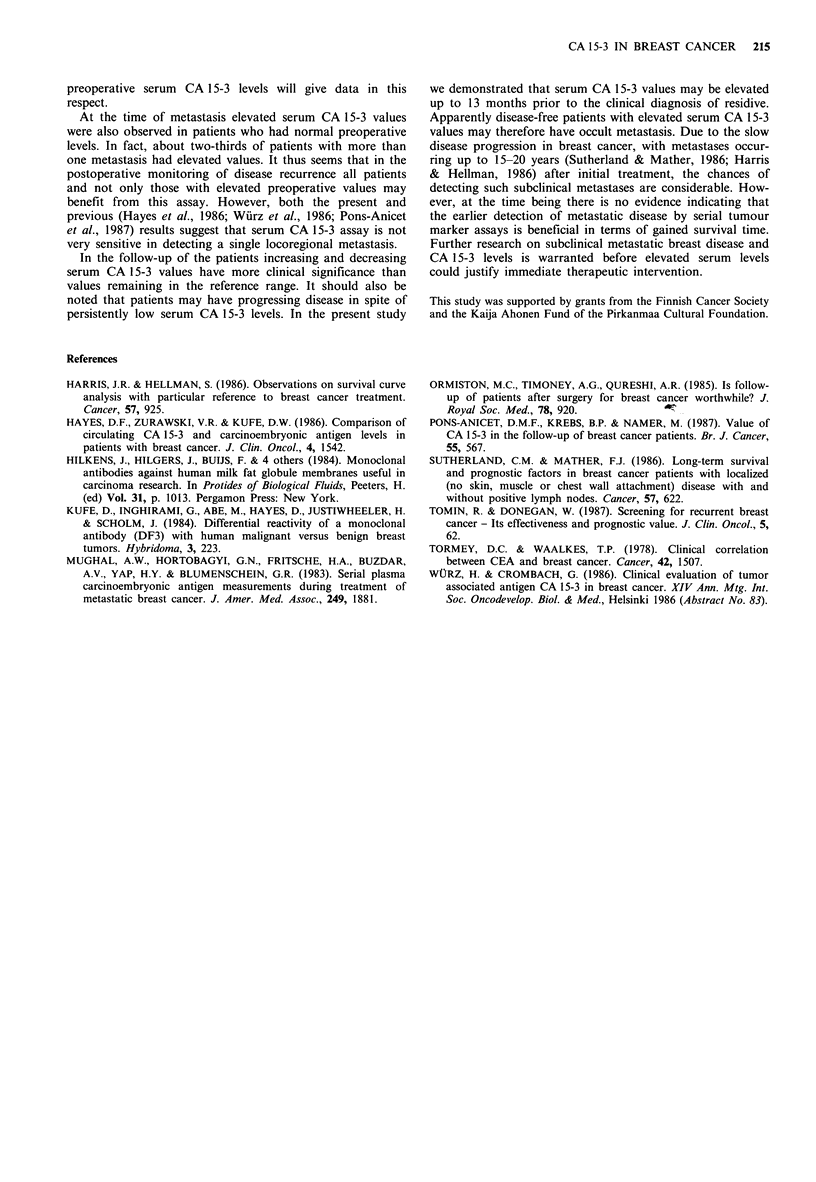


## References

[OCR_00378] Harris J. R., Hellman S. (1986). Observations on survival curve analysis with particular reference to breast cancer treatment.. Cancer.

[OCR_00383] Hayes D. F., Zurawski V. R., Kufe D. W. (1986). Comparison of circulating CA15-3 and carcinoembryonic antigen levels in patients with breast cancer.. J Clin Oncol.

[OCR_00394] Kufe D., Inghirami G., Abe M., Hayes D., Justi-Wheeler H., Schlom J. (1984). Differential reactivity of a novel monoclonal antibody (DF3) with human malignant versus benign breast tumors.. Hybridoma.

[OCR_00400] Mughal A. W., Hortobagyi G. N., Fritsche H. A., Buzdar A. U., Yap H. Y., Blumenschein G. R. (1983). Serial plasma carcinoembryonic antigen measurements during treatment of metastatic breast cancer.. JAMA.

[OCR_00406] Ormiston M. C., Timoney A. G., Qureshi A. R. (1985). Is follow up of patients after surgery for breast cancer worthwhile?. J R Soc Med.

[OCR_00411] Pons-Anicet D. M., Krebs B. P., Mira R., Namer M. (1987). Value of CA 15:3 in the follow-up of breast cancer patients.. Br J Cancer.

[OCR_00416] Sutherland C. M., Mather F. J. (1986). Long-term survival and prognostic factors in breast cancer patients with localized (no skin, muscle, or chest wall attachment) disease with and without positive lymph nodes.. Cancer.

[OCR_00422] Tomin R., Donegan W. L. (1987). Screening for recurrent breast cancer--its effectiveness and prognostic value.. J Clin Oncol.

[OCR_00427] Tormey D. C., Waalkes T. P. (1978). Clinical correlation between CEA and breast cancer.. Cancer.

